# Treatment with Edoxaban Attenuates Acute Stroke Severity in Mice by Reducing Blood–Brain Barrier Damage and Inflammation

**DOI:** 10.3390/ijms22189893

**Published:** 2021-09-13

**Authors:** Michael Bieber, Kathrin I. Foerster, Walter E. Haefeli, Mirko Pham, Michael K. Schuhmann, Peter Kraft

**Affiliations:** 1 Department of Neurology, University Hospital Würzburg, 97080 Würzburg, Germany; bieber_m@ukw.de (M.B.); schuhmann_m@ukw.de (M.K.S.); 2 Department of Clinical Pharmacology and Pharmacoepidemiology, Heidelberg University Hospital, 69120 Heidelberg, Germany; kathrin.foerster@med.uni-heidelberg.de (K.I.F.); walter-emil.haefeli@med.uni-heidelberg.de (W.E.H.); 3 Department of Neuroradiology, University Hospital Würzburg, 97080 Würzburg, Germany; pham_m@ukw.de; 4 Department of Neurology, Klinikum Main-Spessart, 97816 Lohr, Germany

**Keywords:** edoxaban, thrombo-inflammation, blood–brain barrier, tMCAO, experimental stroke, hemorrhagic transformation, NOAC

## Abstract

Patients with atrial fibrillation and previous ischemic stroke (IS) are at increased risk of cerebrovascular events despite anticoagulation. In these patients, treatment with non-vitamin K oral anticoagulants (NOAC) such as edoxaban reduced the probability and severity of further IS without increasing the risk of major bleeding. However, the detailed protective mechanism of edoxaban has not yet been investigated in a model of ischemia/reperfusion injury. Therefore, in the current study we aimed to assess in a clinically relevant setting whether treatment with edoxaban attenuates stroke severity, and whether edoxaban has an impact on the local cerebral inflammatory response and blood–brain barrier (BBB) function after experimental IS in mice. Focal cerebral ischemia was induced by transient middle cerebral artery occlusion in male mice receiving edoxaban, phenprocoumon or vehicle. Infarct volumes, functional outcome and the occurrence of intracerebral hemorrhage were assessed. BBB damage and the extent of local inflammatory response were determined. Treatment with edoxaban significantly reduced infarct volumes and improved neurological outcome and BBB function on day 1 and attenuated brain tissue inflammation. In summary, our study provides evidence that edoxaban might exert its protective effect in human IS by modulating different key steps of IS pathophysiology, but further studies are warranted.

## 1. Introduction

Patients with non-valvular atrial fibrillation (NVAF) are at increased risk of cerebrovascular events, especially if they have already suffered an ischemic stroke (IS) or a transient ischemic attack (TIA) [1]. In these patients, anticoagulation with vitamin K antagonists (VKA) reduced stroke risk by approximately 60% [2]. VKA act through an inhibition of vitamin K-dependent synthesis of coagulation factors in the liver. Their use is complicated by drug–drug and drug–food interactions, the need for regular coagulation monitoring and because of the long half-life of VKA [2]. Meanwhile several non-vitamin K oral anticoagulants (NOAC) have been developed to overcome these limitations. A reliable body of evidence shows that NOAC deliver at least the same protection as VKA in primary and secondary prevention of IS related to NVAF, with approximately half the risk of developing an intracranial hemorrhage (ICH) [3,4,5,6].

Functionally, NOAC act specifically at distinct steps of the plasmatic blood coagulation cascade, either as a direct competitive inhibitor of thrombin (dabigatran) or as factor Xa inhibitors (edoxaban, rivaroxaban, apixaban). Nevertheless, the extent to which off-target effects contribute to stroke protection of NOAC are not fully understood [7].

Edoxaban-targeting coagulation factor Xa is non-inferior to the VKA warfarin with respect to stroke and systemic embolism and is associated with lower bleeding rates [3]. The protective effect might be explained by reduced factor Xa-mediated thrombin activation, because thrombin triggers thrombus formation via activation of fibrinogen and platelets and induces inflammatory processes via protease-activated receptor (PAR) 1, 3, and 4 signaling [8]. Besides various effects of thrombin on the inflammatory environment, thrombin shows a PAR-1/PAR-4-dependent activation of macrophages and microglia [9,10] and triggers the expression of pro-inflammatory cytokines such as tumor necrosis factor alpha (TNF-α) and interleukin-1β (IL-1β) [11], as well as important adhesion molecules, such as vascular cell adhesion molecule-1 (VCAM-1) and intercellular adhesion molecule-1 (ICAM-1), which facilitate leukocyte endothelial transmigration [8]. In addition, thrombin activation leads to a PAR-1-mediated dysfunction of the blood–brain barrier (BBB) [12]. Thus, by inhibiting the activation of thrombin, edoxaban might exert anti-inflammatory and BBB-stabilizing properties in the pathogenesis of IS. 

However, the effect of edoxaban on thrombin-mediated inflammatory processes has not yet been investigated in detail in a model of ischemia/reperfusion injury. Therefore, in the current study we aimed to assess in a clinically relevant experimental model, whether treatment with edoxaban on one hand attenuates IS severity in context with low bleeding risk, and on the other hand has an impact on the local cerebral inflammatory response and BBB function after experimental IS in mice. As VKA have, for decades, been the treatment of choice for the primary and secondary prevention of IS in patients with atrial fibrillation and still are used regularly in patients with contraindications for NOAC, and also because of the different modes of action (specific factor Xa inhibition vs. unspecific inhibition of factors II, VII, IX and X), we additionally wanted to compare the protective effect of VKA vs. edoxaban, and the mechanisms that lead to protection.

## 2. Results

### 2.1. Treatment with Edoxaban Improves Outcome after Stroke in Mice

**Figure 1:**
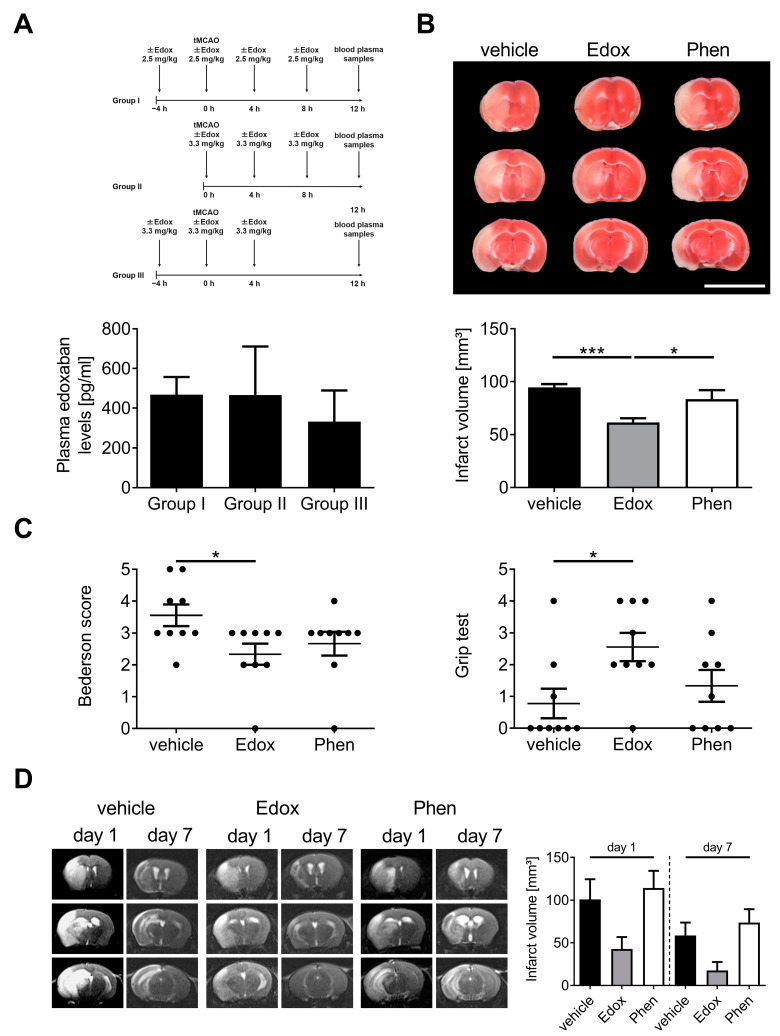
Treatment with edoxaban reduces stroke severity without increasing the risk of intracerebral hemorrhages. (**A**) (**Top**): Definition of the best edoxaban treatment strategy in terms of dose and application time (group I–III) for transient middle cerebral artery occlusion (tMCAO) experiments by measuring plasma edoxaban levels. (**Bottom**): Quantification of edoxaban plasma concentrations of different treatment strategies analyzed by ultra-high-performance liquid chromatography coupled with ultrasensitive tandem mass spectrometer (*n* = 3/group). (**B**) (**Top**): Representative 2,3,5-triphenyltetrazolium chloride (TTC) staining of three corresponding coronal brain sections of vehicle-, edoxaban (Edox)-, or phenprocoumon (Phen)-treated mice euthanized 24 h after tMCAO (scale bar = 10 mm). (**Bottom**): The infarcts (white) appear smallest in the edoxaban group, and this could be confirmed by infarct volumetry (*n* = 9). (**C**) Bederson score (**left**) and grip test (**right**) at day 1 after tMCAO in the three mouse groups indicated above (*n* = 9). (**D**) (**Left**): Serial coronal T2-weighted gradient echo magnetic resonance images (MRI) show extensive hyperintense (bright) ischemic lesions in vehicle- and phenprocoumon-treated mice on day 1 and day 7 after tMCAO, whereas the infarcts were smaller with edoxaban treatment. One representative imaging panel per group is depicted. (**Right**): MRI-based infarct volumetry (*n* = 3–5). * *p* < 0.05, *** *p* < 0.001.

In a first series of experiments, we assessed the best application strategy of edoxaban (in terms of dose and application time) before and after transient middle cerebral artery occlusion (tMCAO). Several strategies are shown in Figure 1A. Thereby, three times oral administration of edoxaban (group II) showed the best combination of high plasma levels and minimal animal stress (regarding number of gavage feedings and duration of the experiment). Thus, this treatment regime was used for all further analyses.

Next, we evaluated the functional role of edoxaban in acute IS. Therefore, we subjected male C57/Bl6 mice to a tMCAO and assessed infarct volumes 23 h after reperfusion. Infarct volumes were significantly smaller in edoxaban-treated mice than in vehicle- or phenprocoumon-treated mice (Figure 1B). Importantly, the smaller infarct volumes of edoxaban-treated mice also translated to a better functional outcome compared to vehicle-treated mice 24 h after stroke induction, using the Bederson score and the grip test (Figure 1C).

Antithrombotic treatment after acute IS is associated with a higher risk of ICH [13]. Therefore, we analyzed the consequences of edoxaban treatment on the dynamics of infarct development by serial magnetic resonance imaging (MRI) in vivo (Figure 1D). Confirming the TTC-based planimetry, the extension of cerebral infarction as delineated by the T2-weighted hyperintense lesion of edoxaban-treated mice was smaller than in vehicle- or phenprocoumon-treated mice 1 day after tMCAO. The stroke protection in edoxaban-treated mice persisted until day 7 after tMCAO as no delayed infarct growth could be detected. MRI experiments showed strong trends towards edoxaban-associated protection at day 1 and 7 compared to vehicle and phenprocoumon, but due to small group sizes (*n* = 3–5), reached no statistical significance. Of note, in all animals, hypointense areas, which typically indicate hemorrhages in T2-weighted images, were absent, supporting the observation that edoxaban treatment does not increase the risk of cerebral hemorrhage compared to the control treatments, even at later stages of infarct development.

### 2.2. Edoxaban Stabilizes the BBB after Stroke

**Figure 2:**
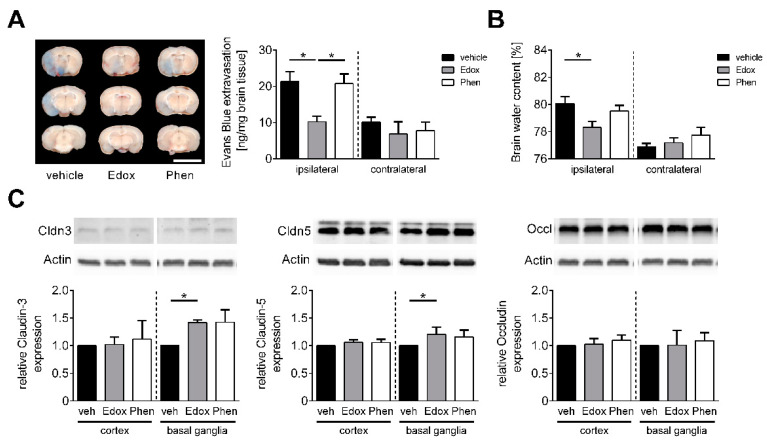
Edoxaban treatment stabilizes the blood–brain barrier and leads to anti-edematous effects in ischemic stroke. (**A**) **Left**: Representative corresponding coronal brain sections of vehicle (veh)-, edoxaban (Edox)- or phenprocoumon (Phen)-treated mice after the injection of the vascular tracer Evans Blue. Brains were analyzed at day 1 after transient middle cerebral artery occlusion (tMCAO) (Scale bar = 10 mm). Vascular leakage was decreased in the cortical and subcortical areas after edoxaban treatment. **Right**: Tissue concentration of Evans Blue in the ischemic (ipsilateral) and contralateral hemispheres of vehicle-, edoxaban-, or phenprocoumon-treated mice 24 h after tMCAO determined by photometry (*n* = 5–8). (**B**) Edema formation as measured by brain water content in the ipsilateral and contralateral hemispheres of vehicle-, edoxaban-, or phenprocoumon-treated mice 24 h after tMCAO (*n* = 4–6). (**C**) **Top**: Claudin (Cldn)-3,-5 and Occludin (Occl) protein expression in the ischemic cortices and basal ganglia of vehicle-, edoxaban-, or phenprocoumon-treated mice at day 1 after tMCAO as determined by immunoblot. Actin was used as loading control. **Bottom**: Densitometric quantification of Cldn-3, -5 and Occl immunoreactivity in the ipsilateral cortex and basal ganglia of vehicle-, edoxaban-, or phenprocoumon-treated mice (*n* = 4). * *p* < 0.05.

One of the main pathophysiological features of IS is disruption of the BBB, which significantly contributes to the development of brain injury and subsequent neurological impairment [14]. Consequently, the extent of BBB damage and the formation of brain edema was assessed after focal cerebral ischemia. Twenty-three h after reperfusion, the integrity of the BBB, as reflected by the concentration of the vascular tracer Evans Blue leaking into the brain parenchyma, was significantly reduced in the ipsilateral but not contralateral hemispheres of edoxaban-treated mice in comparison to vehicle- or phenprocoumon-treated animals (Figure 2A). This observation also correlated with a marked reduction in cerebral water content in the ipsilateral hemispheres of animals treated with edoxaban compared to the other groups (Figure 2B). In accordance with the stabilizing effect of edoxaban at the BBB, augmented levels of the tight junction proteins claudin-3 and claudin-5 were observed by Western blotting in the ischemic basal ganglia of edoxaban-treated mice at day 1 after tMCAO compared with vehicle treatment (Figure 2C). Cortical protein levels as well as levels of occludin were not altered.

### 2.3. Treatment with Edoxaban Reduces Cerebral Inflammation after tMCAO

**Figure 3:**
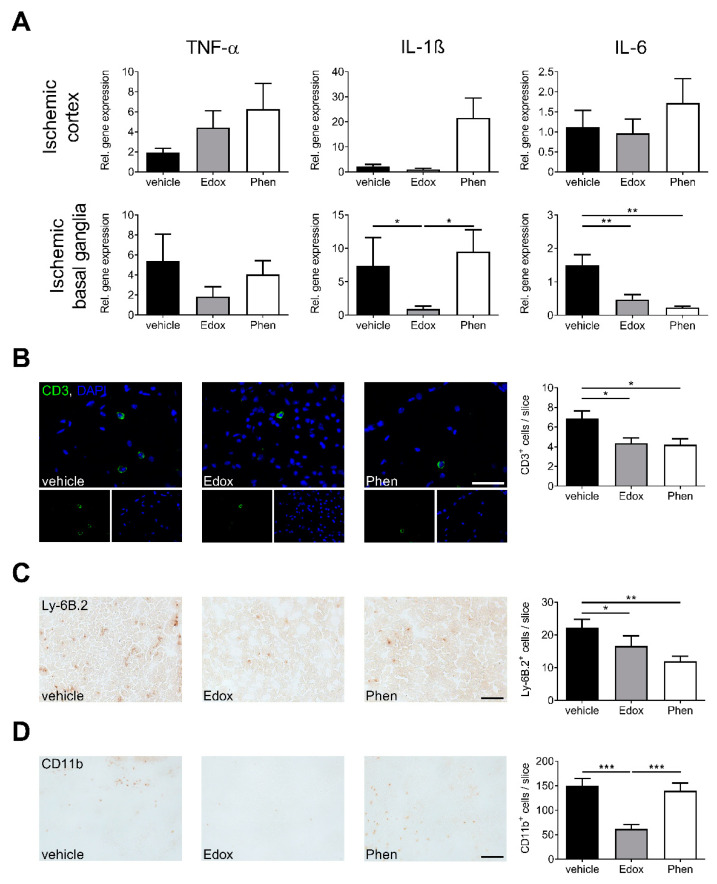
Edoxaban exerts anti-inflammatory effects in ischemic stroke. (**A**) Relative gene expression of tumor necrosis factor alpha (TNFα), interleukin-1β (IL-1β) and interleukin-6 (IL-6) in the ischemic cortices (**top**) and basal ganglia (**bottom**) of vehicle-, edoxaban (Edox)-, or phenprocoumon (Phen)-treated mice 24 h after transient middle cerebral artery occlusion (tMCAO) (*n* = 7–10). (**B**) **Left**: Representative immunohistochemical staining of CD3^+^ T cells (green) and nuclei (blue) in the ischemic hemisphere of vehicle-, edoxaban-, or phenprocoumon-treated mice 24 h after tMCAO (scale bar = 50 µm). **Right**: Quantification of CD3^+^ T cells per slice in the infarcted hemispheres at day 1 after tMCAO (*n* = 5). (**C**,**D**) **Left**: Representative immunocytochemical staining of Ly-6B.2^+^ neutrophils (**C**) and CD11b^+^ macrophages/microglia (**D**) in the ischemic hemisphere of vehicle-, edoxaban-, or phenprocoumon-treated mice 24 h after tMCAO (scale bar = 100 µm). **Right**: Quantification of Ly6B.2^+^ neutrophils and CD11b^+^ macrophages/microglia per slice in the infarcted hemispheres at day 1 after tMCAO (*n* = 5). * *p* < 0.05, ** *p* < 0.01, *** *p* < 0.001.

In line with previous findings of thrombin-induced inflammatory processes via PAR receptors [8] and the functional role of edoxaban on thrombin activation, we analyzed the gene expression profiles of several prototypic proinflammatory cytokines in the brains of vehicle-, edoxaban-, or phenprocoumon-treated mice 24 h after tMCAO (Figure 3A). Thereby, the amount of IL-1β and interleukin-6 (IL-6) mRNA in the ischemic basal ganglia of edoxaban-treated animals was significantly lower compared to vehicle treatment. TNF-α mRNA showed lower gene expression in edoxaban-treated animals than in the other groups without reaching significance. Cortical transcripts did not differ between all tMCAO groups, indicating selective regulation of distinct cytokines.

To assess whether thrombin-induced inflammatory processes and the maintenance of the BBB in edoxaban-treated mice have an effect on the invasion of inflammatory cells into the brain parenchyma [15], we determined the number of CD3^+^ T cells, Ly6b.2^+^ neutrophils, and CD11b^+^ macrophages/microglia in the ischemic hemisphere 24 h after stroke induction (Figure 3B–D). While the numbers of invaded T cells and neutrophils were significantly lower in edoxaban- and phenprocoumon-treated animals in comparison to vehicle treatment, only edoxaban treatment showed a significant reduction in invaded macrophages and microglia compared to the other treatments.

### 2.4. Treatment with Edoxaban Does Not Alter Cellular Adhesion but Reduces Intracerebral Thrombosis

**Figure 4:**
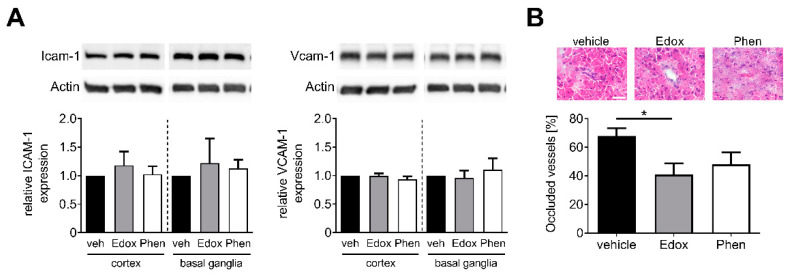
Edoxaban reduces intracerebral thrombosis. (**A**) **Top**: Intercellular adhesion molecule-1 (ICAM-1) and vascular cell adhesion molecule-1 (VCAM-1) protein expression in the ischemic cortices and basal ganglia of vehicle (veh)-, edoxaban (Edox)-, or phenprocoumon (Phen)-treated mice at day 1 after tMCAO as determined by immunoblot. Actin was used as loading control. **Bottom**: Densitometric quantification of ICAM-1 and VCAM-1 immunoreactivity in the ipsilateral cortex and basal ganglia of vehicle-, edoxaban-, or phenprocoumon-treated mice (*n* = 4). (**B**) **Top**: Representative H&E stains in the ischemic hemisphere of vehicle-, edoxaban-, or phenprocoumon-treated mice 24 h after tMCAO (Scale bar = 50 µm). Thrombotic vessels were shown in vehicle- and phenprocoumon-treatment groups, whereas an open vessel is shown for the edoxaban group. **Bottom**: Percentage of occluded vessels in the ischemic hemispheres of vehicle-, edoxaban-, or phenprocoumon-treated mice at day 1 after tMCAO (*n* = 5). * *p* < 0.05.

Finally, we aimed to examine whether edoxaban treatment has an effect on the regulation of endothelial adhesion molecules or on the number of intracerebral thromboses. Importantly, neither edoxaban nor phenprocoumon treatment altered the expression levels of endothelial adhesion molecules ICAM-1 and VCAM-1 at day 1 after tMCAO (Figure 4A). Nevertheless, only edoxaban-treated mice showed a significant decrease in occluded ipsilateral cerebral microvessels compared with the vehicle group 24 h after ischemia induction (Figure 4B).

## 3. Discussion

In this study, we show that therapeutic treatment with edoxaban improves outcomes after focal cerebral ischemia in mice. One day after tMCAO, animals treated with edoxaban developed smaller infarct volumes compared to animals treated with vehicle or phenprocoumon. In addition, functional tests yielded smaller motor deficits in mice receiving edoxaban compared to vehicle-treated animals. These findings are in agreement with other preclinical studies, showing that NOAC-treated animals develop milder stroke phenotypes in the tMCAO model compared to control animals [7,16]. In line with this, a study in humans also revealed that patients treated with NOAC exhibited less neurologic symptoms, as assessed by the National Institutes of Health Scale score [17], on admission and a more favorable outcome at discharge compared to those without anticoagulants [18]. 

These findings are in accordance with the results of previous studies on rats undergoing tMCAO during rivaroxaban and dabigatran etexilate treatment, in which infarct volumes of treated animals were also significantly smaller than those of vehicle-treated rats [7,16]. Moreover, in the current study, we could reproduce former results [7,16], proving that anti-inflammatory and BBB-preserving properties of NOAC might play an important protective role in experimental IS. This can in part be explained by intertwined mechanisms of thrombotic and pro-inflammatory pathways (‘thrombo-inflammation’), which can facilitate further infarct development [19,20,21]. 

Of note, edoxaban treatment increased the expression of important tight junction proteins and reduced pro-inflammatory cytokines in the basal ganglia, but not the cortices. As edoxaban-treated animals regularly showed basal ganglia infarctions without cortical involvement, we assume that edoxaban protects the BBB function specifically in ischemic areas of the brain. This might contribute to the reduced number of immune cells invading the basal ganglia and could consequently lead to a reduced expression of pro-inflammatory cytokines. Despite unchanged infarct volumes, phenprocoumon treatment also exerted an anti-inflammatory effect (Figure 3) but did not preserve the BBB (Figure 2).

Application time of edoxaban might play a crucial role, especially the question of whether treatment starts before (prophylactically) or after tMCAO (therapeutically). While other studies focused on prophylactic treatment [7,16,22], in our investigation we analyzed the efficacy of edoxaban in a therapeutic approach. Edoxaban was given immediately after withdrawal of the occluding filament out of the middle cerebral artery (1 h after initiation of tMCAO), resembling the clinical situation of a patient with acute IS receiving endovascular mechanical thrombectomy at the earliest possible time point after intracranial vessel occlusion. Although edoxaban concentrations were approximately 400-fold below the concentrations observed in patients, the experimental setting has high translational validity [23]. The results indicate that the treatment of cerebral ischemia may require being lower than the currently approved edoxaban doses, but the concentration-effect relationship will necessitate proper further assessment.

Importantly, in a serial MRI-analysis up to 1 week after tMCAO, we found no increased risk of ICH or hemorrhagic transformation in mice treated with edoxaban. This finding supports observations available from experimental stroke models investigating dabigatran [7,24,25] and rivaroxaban [16,26,27]. In humans, intravenous thrombolysis is contraindicated for patients on NOAC. Nevertheless, in case reports and case series, successful thrombolytic treatment in patients under NOAC therapy without bleeding complications have been described [28,29,30]. Hence, experimental and clinical data suggest that NOAC administration provides a safe treatment option for primary and secondary stroke prevention.

In contrast to our previous study investigating dabigatran etexilate [7], in the present study, edoxaban prevented the disruption of the BBB, determined 24 h after IS as measured with Evans Blue extravasation, cerebral water content, and claudin-3 and -5 expression. It is known that edoxaban also affects thrombin and thrombin receptor-activating peptide (TRAP)-induced platelet aggregation [31,32]. Therefore, the finding of BBB stabilization is in agreement with a study reporting that PAR-4-deficient mice exhibited a significant attenuation of cerebral edema compared to wild-type mice [33], which emphasizes the significance of thrombin in edema formation. As the effect of thrombin on BBB disruption depends on its concentration, we assume that the thrombin concentration in edoxaban-treated animals is reduced, thus leading to a reduction in thrombin/PAR-4-mediated breakdown of the BBB.

## 4. Materials and Methods

### 4.1. Animals

We randomized male C57Bl/6 N mice (6–8 weeks old) and subjected them to tMCAO [34]. All animal experiments were approved by the responsible local animal care committees (Regierung von Unterfranken) and were conducted in accordance with the US National Institutes of Health Guide for the Care and Use of Laboratory Animals. The experiments were designed, performed and reported according to the Animal Research: Reporting of In Vivo Experiments guidelines [35]. All mice were purchased from Charles River Laboratories (Sulzfeld, Germany).

### 4.2. Animal Treatment

Edoxaban tosilate hydrate was provided from Daiichi Sankyo Co., Ltd. (Tokyo, Japan). Two different doses of edoxaban (2.5/3.3 mg/kg body weight, oral gavage via gastric tube, dissolved in 0.5% (*w*/*v*) methyl cellulose) were administered in 3 different dosing regimens, as shown in Figure 1A. For tMCAO experiments, edoxaban (3.3 mg/kg body weight, dissolved in 0.5% (*w*/*v*) methyl cellulose) or vehicle (0.5% (*w*/*v*) methyl cellulose) were given as oral gavage via a gastric tube as mentioned for group 2 in Figure 1A. The VKA phenprocoumon (0.3 mg/kg body weight, oral gavage via gastric tube, dissolved in 0.5% (*w*/*v*) methyl cellulose) was administered 3 times (72, 48, and 24 h) before tMCAO to reach international normalized ratios (INR) between 2–3 at the time of tMCAO.

### 4.3. Measurement of Edoxaban Plasma Concentrations

To measure edoxaban plasma concentration, mice were sacrificed, and blood from the left ventricle was sampled in lithium heparin tubes. The lithium heparin sample tubes were gently inverted 8–10 times to ensure thorough mixing of blood and anticoagulants. Within 30 min of blood collection, the samples were centrifuged for 10 min at 2000× *g* and 20 °C. The harvested plasma samples were stored at −80 °C until further analysis. Plasma concentrations of edoxaban were analyzed by ultra-high-performance liquid chromatography coupled with an ultrasensitive tandem mass spectrometer (Waters Xevo TQ-S, Waters, Eschborn, Germany). Plasma samples (100 µL) were quantified according to a published UPLC-MS/MS assay [36]. A linear calibration range was present between 2.5 to 2000 pg/mL. Within this range, edoxaban concentrations were quantified with accuracy and precision values below 15%. The lower limit of quantification was 2.5 pg/mL. The assay fulfilled the guidelines on bioanalytical method validation from the US Food and Drug Administration (FDA) [37] and European Medicines Agency (EMA) [38]. 

### 4.4. Ischemia Model

Focal cerebral ischemia was induced by 60 min tMCAO exactly as described previously [34]. Edema-corrected stroke volumes were assessed 24 h and 7 d after tMCAO, based on 2,3,5-triphenyltetrazolium chloride (TTC) staining or serial MRI. Sample size calculation was performed using the stroke volumes from previous studies [19,39,40], a standard deviation of 20% to the respective mean values, a power of 80% and a probability of a type I error of <5%. Therefore, a group size ≥ 7 was necessary to reliably detect a 30% difference in stroke volume.

### 4.5. Exclusion Criteria

Mice were excluded from endpoint analyses for the following pre-specified reasons: (1) death before predefined experimental endpoint; (2) drop-out score (weight loss, general condition, spontaneous behavior); (3) operation time > 10 min (to exclude the influence of prolonged anesthesia and increase group comparability).

### 4.6. Assessment of Functional Outcome

Global neurological deficits were quantified according to the Bederson score [41]. To monitor function and coordination, the grip test was used [41,42]. Neurological outcome was assessed 24 h after stroke.

### 4.7. Triphenyltetrazolium Chloride (TTC) Staining

Infarct size was analyzed by vital staining using 2% (wt/vol) TTC in phosphate buffer. Edema-corrected infarct volumes were calculated by planimetry (ImageJ software, National Institues of Health, Bethesda, MD, USA) [34].

### 4.8. Magnetic Resonance Imaging

To analyze infarct dynamics and to scan for possible intracerebral bleeding, stroke assessment was performed by serial MRI at 3.0 T (MAGNETOM Trio, SIEMENS, Erlangen, Germany) at day 1 and day 7 after tMCAO [34]. The image protocol comprised a coronal multi-slice TSE (TR/TE (repetition time/echo time) = 2100/113 ms, 18 slices, BW (bandwidth) = 120 Hz/px, in-plane resolution: 0.17 × 0.17 × 1.0 mm^3^) as well as a 3D CISS (TR/TE = 11.57/4.87 ms, BW = 130 Hz/px, in-plane resolution: 0.25 × 0.25 × 0.5 mm^3^, FA (flip angle) = 70°) for T2-weighted imaging. MRIs were visually assessed by researchers blinded to the prior treatment with respect to infarct morphology and, in particular, the occurrence of ICH [39].

### 4.9. Determination of BBB Leakage and Brain Edema Formation

To determine the permeability of the cerebral vasculature 100 μL of 2% Evans Blue tracer (Sigma Aldrich, Taufkirchen, Germany) diluted in 0.9% NaCl was i.v. injected 1 h after the induction of tMCAO [19]. After 24 h, brains were removed and cut into three 2-mm thick coronal slices. In the dark, brain slices were fixed in 4% PFA and afterwards, all three slices were cut into pieces and transferred into Eppendorf cups. After adding 500 μL of formamide and incubation for 24 h at 50 °C in the dark, tubes were centrifuged for 20 min at 16,000× *g*. Fifty μL of the supernatant was transferred to a 96-well plate and fluorescence intensity was measured in duplicate by a microplate fluorescence reader (INFINITE 200 Pro, TECAN, Männedorf, Switzerland) with an excitation at 620 nm and an emission at 680 nm. The tissue concentration for each sample was calculated from the standard curve using linear regression analysis.

To assess the extent of brain edema, 24 h after tMCAO induction, mice were sacrificed, brains were removed, hemispheres separated and weighed to assess the wet weight (WW) [34]. Subsequently, the hemispheres were dried for 72 h at 60 °C and the dry weight (DW) was determined. Hemispheric water content (%) was calculated using the following formula: ((WW − DW)/WW) × 100 [34].

### 4.10. Protein Extraction and Western Blot Analysis

Protein extraction and Western blot analysis were performed according to standard procedures using antibodies against claudin-3 (1:150, 34–1700, Thermo Fisher Scientific, Waltham, MA, USA), claudin-5 (1:250, sc-28670, Santa Cruz Biotechnology, Dallas, TX, USA), occludin (1:250, 611090, BD Biosciences, San Jose, CA, USA), ICAM-1 (1:250, sc1511, Santa Cruz Biotechnology, Dallas, TX, USA) VCAM-1 (1:2000, ab134047, Abcam, Cambridge, UK) and actin (1:500,000, A5441, Merck, Darmstadt, Germany) [34].

### 4.11. Quantitative Real-Time PCR

Quantitative real-time polymerase chain reaction was performed as described previously [43]. The following primers were purchased from Thermo Fisher Scientific (Waltham, MA, USA): interleukin 1β (IL-1β; Mm00434228_m1), interleukin-6 (IL-6; Mm00446190_m1), tumor necrosis factor α (TNF-α; Mm00443258_m1) and glyceraldehyde 3-phosphate dehydrogenase (GAPDH, Taq-Man Predeveloped Assay Reagents for gene expression, part number: 4352339E). GAPDH was used as an endogenous control.

### 4.12. Histology and Immunohistochemistry

Histology and immunohistology of cryo-embedded brain sections were performed using anti-CD3 (100212, BioLegend, San Diego, CA, USA), anti-CD11b (MCA711, Bio-Rad, Hercules, CA, USA) and anti-Ly6b.2 (MCA771G, Bio Rad, Hercules, CA, USA) antibodies as described previously [34,44]. For all quantifications, identical brain sections at the level of the basal ganglia (0.5 mm anterior from bregma) were selected and cell counting over the whole hemisphere was performed from 5 subsequent slices of 5 different animals under a microscope (Leica DMi8 equipped with the DMC 2900/DFC 3000 G camera control and LAS X software (Leica, Wetzlar, Germany)). Negative controls for all histological experiments included omission of primary or secondary antibody and gave no signals (not shown). To depict occluded vessels, we performed hematoxylin-eosin staining of representative brain slices and calculated the percentage of occluded vessels within all vessels of 5 slices of 5 different animals of each group with the aforementioned microscope. All shown pictures were recorded in the peri-infarct or the core region.

### 4.13. Statistical Analysis

All results are expressed as mean ± SEM. For statistical analysis, the GraphPad Prism 6 software package (GraphPad Software, San Diego, CA, USA) was used. Data were tested for Gaussian distribution with the D’Agostino–Pearson omnibus normality test and then analyzed by 1-way analysis of variance (ANOVA) with post-hoc Bonferroni adjustment for *p* values or for nonparametric analysis compared by Kruskal–Wallis test with post-hoc Dunn’s corrections. Probability values < 0.05 were considered to indicate statistically significant results.

## 5. Conclusions

In this study, we demonstrated that therapeutic treatment with edoxaban decreases stroke volumes and improves functional outcome after focal cerebral ischemia in mice without an increase in the rate of ICH. In this process, anti-inflammatory and BBB-preserving properties of edoxaban contribute to the protective effect after stroke. Our study provides experimental data that treatment with edoxaban might also be beneficial when applied in a therapeutic strategy in the context of acute IS. Nevertheless, further basic science and clinical studies are warranted.

## Data Availability

The analyzed data sets generated during the study are available from the corresponding author on reasonable request.
